# Persisting disparities between sexes in outcomes of ruptured abdominal aortic aneurysm hospitalizations

**DOI:** 10.1038/s41598-017-18451-2

**Published:** 2017-12-21

**Authors:** Mark Stuntz, Céline Audibert, Zheng Su

**Affiliations:** Deerfield Institute, New York, NY USA

## Abstract

We sought to describe and analyze discrepancies between sexes in the outcomes of patients hospitalized for ruptured abdominal aortic aneurysms (rAAA) by conducting a retrospective analysis of the Nationwide Inpatient Sample. The review included all adult patients (≥18 years old) hospitalized with a primary diagnosis of rAAA between January 2002 and December 2014. In-hospital mortality differences between females and males were analyzed overall and separately among those receiving endovascular AAA repair (EVAR) or open AAA repair (OAR). In-hospital mortality for females declined from 61.0% in 2002 to 49.0% in 2014 (P for trend <0.001), while mortality for males declined from 48.6% in 2002 to 32.2% in 2014 (P for trend <0.001). Among those receiving EVAR, females were significantly more likely to die in the hospital than males (adjusted odds ratio [OR], 1.44; 95% CI, 1.12–1.84). In addition, the odds of mortality among those receiving OAR were higher for females than males (adjusted OR, 1.14; 95% CI: 1.00–1.31). These data provide evidence that despite overall decreasing trends in mortality for both sexes, females remain at higher risk of death compared with males regardless of surgical repair procedure.

## Introduction

Abdominal aortic aneurysm (AAA) affects approximately 4–5% of adults over 50 years of age, and is several times more common in men than in women^1–3^. Mortality among patients with AAA is high, primarily due to ruptured abdominal aortic aneurysm (rAAA)^4^. According to a systematic review and meta-analysis, 32% of patients with rAAA die before reaching the hospital^5^, and those that do survive long enough to reach medical care have been shown to have a 39% in-hospital mortality rate^6^.

Females with AAA are more likely to present at older age with rAAA and have higher risk of mortality compared with males^7–9^. Several reviews have found that females are less likely to undergo surgical treatment of rAAA^10–12^, and it has been postulated that the diagnosis of rAAA in females may be delayed and a sex bias may exist in selecting candidates for surgery^13^.

Females with rAAA tend to be underdiagnosed as well as undertreated, which may be a factor in having worse prognoses. A 10-year inpatient sample study from the Centers for Medicare and Medicaid Services showed the average mortality rate for females with rAAA was 52.8% compared with 44.2% among males^14^. In a report on national rAAA outcomes from 2001 to 2004, a higher percentage of females with AAA presented with rupture compared with males, and this trend did not decrease over time^15^.

Despite this wealth of existing information, there is a need for a definitive examination of disparities in rAAA outcomes between females and males in the United States. To that end we have utilized 13 years of all-payer inpatient data, aimed at describing and quantifying trends in rAAA hospitalizations and disparities in outcomes between sexes.

## Methods

### Data Source

This study utilized the 2002–2014 discharge data from the Nationwide Inpatient Sample (NIS), provided by the Agency for Healthcare Research and Quality’s (AHRQ) Healthcare Cost and Utilization Project (HCUP)^16^. The NIS is the largest publicly-available all-payer inpatient database in the United States. Each year of data contains information from more than 7 million hospitalizations, representing a 20-percent stratified sample of all US inpatient stays in nonfederal hospitals. Included sampling weights were used to calculate national estimates.

This study was approved by the Deerfield Institute Research Review Committee and deemed to be in full compliance of HIPAA (Health Insurance Portability and Accountability Act) guidelines, as it was conducted with publicly available deidentified data.

### Study Population and Variables

We identified all hospitalizations among adults ≥18 years or older between January 2002 and December 2014 with a primary diagnosis of rAAA using *International Classification of Diseases, Ninth Edition, Clinical Modification (ICD-9-CM)* codes 441.3, 441.5, or 441.6. Endovascular AAA repair (EVAR) procedures were identified using ICD-9-CM code 39.71 and open AAA repair (OAR) procedures using codes 38.34, 38.44, 38.64, and 39.52. Perioperative complications were identified using ICD-9-CM codes for acute myocardial infarction (410.01, 410.11, 410.21, 410.31, 410.41, 410.51, 410.61, 410.81), acute renal failure (584.0, 584.5, 584.6, 584.7, 584.8, 584.9), venous thromboembolism (415.11–415.19, 451.11–451.19, 451.81, 451.83, 453.2, 453.40–453.42, 671.30–671.33, 671.40–671.44, 673.20–673.24), gastrointestinal (GI) bleed (578.0, 578.1, 578.9), and bowel obstruction without mention of hernia (560.0–560.2, 560.30–560.32, 560.39, 560.81, 560.89, 560.9). These perioperative complications were selected *a priori* based on their established clinical significance and relevance to outcomes in patients with rAAA^17^.

Patient characteristics were extracted from the database, including demographics (age, sex, race, expected primary payer, and income quartile for patient’s ZIP code), hospital characteristics (US geographic region, location and teaching status, and hospital bed size), and 29 Elixhauser comorbidities. The Elixhauser comorbidity index is a validated measure of comorbidities in large administrative databases as defined by AHRQ^18^. We used the van Walraven modification of the Elixhauser comorbidity measures to calculate a single numeric score for disease burden^19^.

### Outcomes

Our primary outcomes were sex disparities among national temporal trends in rate of hospitalizations, mean charges per hospitalization, in-hospital mortality, and perioperative complications following rAAA repair.

### Hospital Charges

Hospital charges included in the NIS are the amount the hospital billed to Medicare, Medicaid, private insurance, and other sources for the entire hospital stay. Mean hospital charges reported have been adjusted to 2014 dollars using the Bureau of Labor Statistics’ inflation calculator.

### Statistical Analyses

National weighted estimates were obtained by applying trend weights provided by AHRQ and were used for all statistical analyses. Descriptive statistics were reported as weighted values with mean (standard deviation [SD]) or count with percentages, as appropriate.

National incidence rates of hospitalizations for rAAA were calculated by dividing the annual weighted number of hospitalizations for each sex by the appropriate adult population on July 1 using estimates from the US Census Bureau’s American FactFinder database^20^, and expressing the result as rate of hospitalizations per 100,000 adults. Univariate between-group differences were analyzed using Pearson χ^2^ tests for categorical variables and 2-tailed *t* tests for continuous variables. The Cochran-Armitage test was used for temporal trend analysis of categorical variables and one-way ANOVA was used to assess the significance of differences between means of continuous variables.

Multivariable logistic regression was used to examine differences in in-hospital mortality and perioperative outcomes between females and males. Covariates in the regression models included patient characteristics, comorbidities, and hospital characteristics. All patients were included in the regression models except those with missing data on age, race, expected primary payer, income quartile, and Elixhauser comorbidities.

All analyses were performed with SPSS Complex Samples module version 23.0 (IBM Corp., Armonk, NY) with statistical significance defined as P ≤ 0.05. Complex sample data analysis adjusts for weights, cluster, and stratification of the sampling design to produce unbiased national estimates of population means and frequencies from the sample after taking into account weights for over- or under-sampling of specific groups^21^. This complex sample analysis method is comparable with a multilevel mixed-effects model with random effects for hospital^22^. The Taylor-series linearization method was used to calculate standard errors^23^.

## Results

### Characteristics of rAAA Hospitalizations

A total of 75,698 weighted records with a primary diagnosis of rAAA were identified in the NIS database from 2002 to 2014. Table 1 summarizes the differences between females and males in patient characteristics, procedure characteristics, and hospital characteristics. Of the 75,698 total hospitalizations examined, 22,177 (29.3%) were females and 53,521 (70.7%) were males. There were significant differences between females and males relating to age, race/ethnicity, expected primary payer, hospital geographic region, hospital location and teaching status, and hospital bed size. Females had significantly shorter mean lengths of stay, 8.2 days (13.2), compared with males, 10.5 days (13.9) (P < 0.001). There were no significant differences in calculated van Walraven Elixhauser comorbidity score between females, 5.4 (5.4), and males, 5.5 (5.6) (P = 0.24).

**Table 1:** Descriptive characteristics of patients hospitalized with primary diagnosis of rAAA, 2002–2014.

	Males (n = 53,521)	Females (n = 22,177)	P Value
**Patient characteristics, No. (%)**			
Mean age, years (SD)	73.4 (10.1)	78.2 (9.8)	<0.001
Age group, y			
18–54	1,623 (3.0)	428 (1.9)	<0.001
55–64	8,730 (16.3)	1,455 (6.6)	
65–74	17,937 (33.5)	5,176 (23.3)	
75–84	17,493 (32.7)	8,860 (39.9)	
85+	7,739 (14.5)	6,257 (28.2)	
Race/ethnicity^a^			
White	36,292 (86.3)	14,729 (84.1)	<0.001
African-American	2,110 (5.0)	1,351 (7.7)	
Hispanic	1,629 (3.9)	605 (3.5)	
Asian or Pacific Islander	805 (1.9)	363 (2.1)	
Native American	120 (0.3)	53 (0.3)	
Other	1,086 (2.6)	405 (2.3)	
Expected primary payer^a^			
Medicare	38,838 (72.7)	18,558 (83.8)	<0.001
Medicaid	1,405 (2.6)	550 (2.5)	
Private including HMO	10,104 (18.9)	2,379 (10.7)	
Self-pay	1,714 (3.2)	412 (1.9)	
No charge	111 (0.2)	18 (0.1)	
Other	1,272 (2.4)	230 (1.0)	
Income quartile for patient’s ZIP code^a^			
0–25th percentile	11,636 (22.3)	4,900 (22.6)	0.42
26th-50th percentile	14,087 (27.0)	6,037 (27.8)	
51st-75th percentile	13,466 (25.8)	5,660 (26.1)	
76th-100th percentile	12,927 (24.8)	5,109 (23.5)	
Comorbidities			
Mean van Walraven Elixhauser score (SD)	5.5 (5.6)	5.4 (5.4)	0.24
Mean length of stay, days (SD)	10.5 (13.9)	8.2 (13.2)	<0.001
Procedure characteristics			
EVAR	10,334 (19.3)	3,092 (13.9)	<0.001
OAAR	30,612 (57.2)	9,831 (44.3)	<0.001
No rAAA repair	12,575 (23.5)	9,254 (41.7)	<0.001
**Hospital characteristics**			
Hospital US geographic region			
Northeast	10,087 (18.8)	5,329 (24.0)	<0.001
Midwest	14,196 (26.5)	5,864 (26.4)	
South	18,550 (34.7)	7,058 (31.8)	
West	10,688 (20.0)	3,927 (17.7)	
Hospital location and teaching status^*a*^			
Rural	4,745 (8.9)	2,340 (10.6)	0.008
Urban, non-teaching	19,430 (36.4)	7,978 (36.1)	
Urban, teaching	29,133 (54.7)	11,807 (53.4)	
Hospital bed size^*a,b*^			
Small	4,263 (8.0)	2,167 (9.8)	0.001
Medium	11,648 (21.9)	4,979 (22.5)	
Large	37,397 (70.2)	14,980 (67.7)	

^a^Data for race/ethnicity were missing for 21.3% of records, expected primary payer for 0.1% of records, income quartile for 2.5% of records, and hospital characteristics for 0.3% of records.

^b^Hospital bed size categories are specific for location and teaching status.

Among females, 13.9% of hospitalizations received EVAR compared with 19.3% among males (P < 0.001). Female hospitalizations also had a significantly lower proportion receive OAR compared with males (44.3% vs 57.2%, respectively; P < 0.001). A significantly higher proportion of females compared with males did not receive any coded rAAA repair procedure (41.7% vs 23.5%, P < 0.001).

### Trends in Rate of rAAA Hospitalizations and Mean Charges

**Figure 1:**
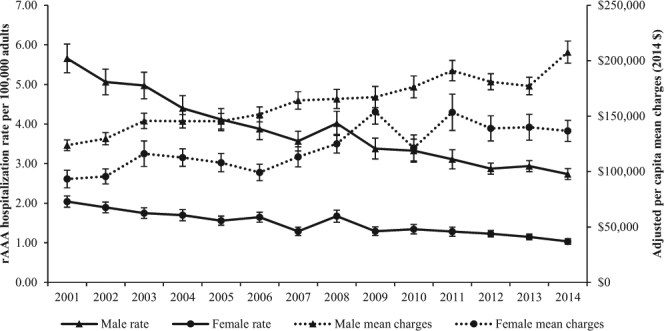
Temporal trends in rate of hospitalizations per 100,000 adults and adjusted mean charges for stays with primary diagnosis of rAAA. Mean hospitalization charges for each year have been adjusted to 2014 inflation dollars. Error bars indicate SE.

Relative to the underlying population, the rate of hospitalizations for rAAA among females decreased from 1.90 per 100,000 in 2002 to 1.04 per 100,000 in 2014 (P for trend <0.001) (Fig. 1). Adjusted mean hospital charges (in 2014 $) for females increased from $95,639 in 2002 to $136,694 in 2014 (P for trend <0.001). The hospitalization rate for males decreased from 5.06 per 100,000 in 2002 to 2.74 per 100,000 in 2014 (P for trend <0.001), while adjusted mean hospital charges increased from $129,717 in 2002 to $207,725 in 2014 (P for trend <0.001).

### Perioperative Complications and In-Hospital Mortality

**Figure 2:**
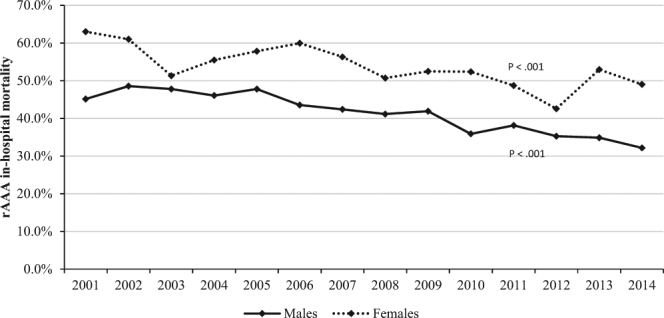
Temporal trends for rAAA in-hospital mortality among males and females.

In the overall study cohort, 53.5% of females with rAAA died in the hospital compared with 41.9% of males (P < 0.001). In-hospital mortality for females declined from 61.0% in 2002 to 49.0% in 2014 (P for trend <0.001), while mortality for males declined from 48.6% in 2002 to 32.2% in 2014 (P for trend <0.001) (Fig. 2).

Among patients receiving EVAR, 30.5% of females died in the hospital compared with 23.5% of males (P < 0.001). After adjusting for covariates, females receiving EVAR had 44% higher odds of in-hospital mortality than males receiving EVAR (adjusted OR, 1.44; 95% CI, 1.12–1.84) (Table 2). There were no significant differences between females and males in the odds of perioperative complications among those receiving EVAR, after adjusting for covariates.

**Table 2:** In-hospital outcomes of patients undergoing repair for rAAA.

	Males	Females	P Value	Odds Ratio, Females vs Males (95% CI)			
				Unadjusted	Adjusted^a^	P Value^b^	R^2^ Value^c^
EVAR, No. (%)	n = 10,334	n = 3,092					
In-hospital mortality	2,431 (23.5)	940 (30.5)	<0.001	1.43 (1.17–1.73)	1.44 (1.12–1.84)	0.004	0.22
Perioperative complications							
Acute myocardial infarction	110 (1.1)	15 (0.5)	0.18	0.44 (0.13–1.50)	0.79 (0.19–3.23)	0.74	0.28
Acute renal failure	3,339 (32.3)	964 (31.2)	0.59	0.95 (0.79–1.14)	0.81 (0.63–1.05)	0.11	0.39
Venous thromboembolism	222 (2.2)	83 (2.7)	0.43	1.25 (0.72–2.16)	1.24 (0.66–2.35)	0.50	0.11
GI bleed	237 (2.3)	55 (1.8)	0.44	0.77 (0.40–1.48)	0.71 (0.32–1.57)	0.39	0.19
Bowel obstruction without mention of hernia	1,079 (10.4)	222 (7.2)	0.01	0.66 (0.48–0.92)	0.75 (0.52–1.10)	0.14	0.16
OAR, No. (%)	n = 30,612	n = 9.831					
In-hospital mortality	11,731 (38.4)	4,493 (45.7)	<0.001	1.35 (1.22–1.49)	1.14 (1.00–1.31)	0.05	0.20
Perioperative complications							
Acute myocardial infarction	491 (1.6)	124 (1.3)	0.27	0.78 (0.51–1.21)	0.98 (0.57–1.67)	0.94	0.11
Acute renal failure	12,113 (39.6)	3,668 (37.3)	0.06	0.91 (0.82–1.00)	0.90 (0.78–1.03)	0.12	0.25
Venous thromboembolism	802 (2.6)	157 (1.6)	0.008	0.60 (0.42–0.88)	0.45 (0.28–0.72)	0.001	0.13
GI bleed	865 (2.8)	300 (3.1)	0.59	1.08 (0.81–1.45)	1.20 (0.85–1.69)	0.30	0.18
Bowel obstruction without mention of hernia	4,529 (14.8)	908 (9.2)	<0.001	0.59 (0.50–0.69)	0.58 (0.47–0.70)	<0.001	0.16

^a^Adjusted for patient characteristics, comorbidities, and hospital characteristics.

^b^P values reported for adjusted ORs.

^c^Cox and Snell R^2^ values reported for adjusted models.

Among patients receiving OAR, 45.7% of females died in the hospital compared with 38.4% of males (P < 0.001). The results of the logistic regression model showed that females receiving OAR had 14% higher odds of in-hospital mortality than males receiving OAR (adjusted OR, 1.14; 95% CI, 1.00–1.31). Females receiving OAR were less likely than males to experience certain perioperative complications, including venous thromboembolism (adjusted OR, 0.45; 95% CI, 0.28–0.72) and bowel obstruction without mention of hernia (adjusted OR, 0.58; 95% CI, 0.47–0.70).

**Figure 3:**
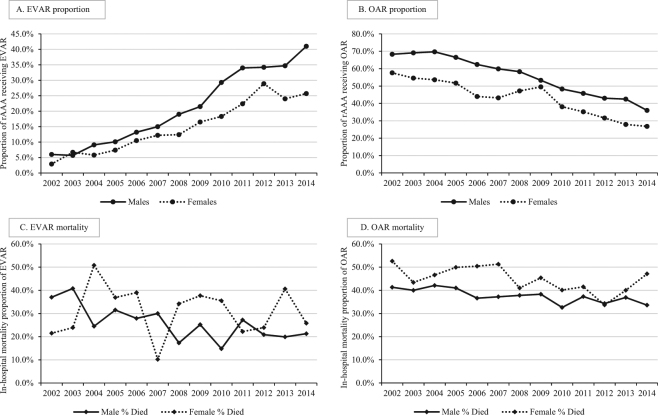
Temporal trends for rAAA surgical repair utilization and mortality. (**A**) There were significant (P for trend <0.001) increasing trends for both males and females in utilization of EVAR; (**B**) significant (P for trend <0.001) decreasing trends for both males and females in utilization of OAR; (**C**) significant (P for trend <0.001) decreasing trend in mortality among males receiving EVAR, and no significant change among females (P for trend =0.51); (**D**) significant (P for trend <0.001) decreasing trend in mortality among males receiving OAR, and no statistically significant change among females (P for trend =0.06).

Temporal trends in surgical repair utilization and mortality are depicted in Fig. 3. There were significant increasing trends for both females and males in the proportion of rAAA hospitalizations that received EVAR, with concomitant significant decreasing trends for both sexes in the proportion receiving OAR (P for trend <0.001 for both females and males, EVAR and OAR). The in-hospital mortality proportion among females receiving EVAR varied substantially throughout the study period and no significant trend was observed (P for trend = 0.51), while the in-hospital mortality proportion among males receiving EVAR decreased significantly (P for trend <0.001). Among patients receiving OAR, there was a decreasing trend in mortality for females, though it was not statistically significant (P for trend = 0.06), while mortality among males significantly decreased from 41.3% in 2002 to 33.6% in 2014 (P for trend <0.001).

**Figure 4:**
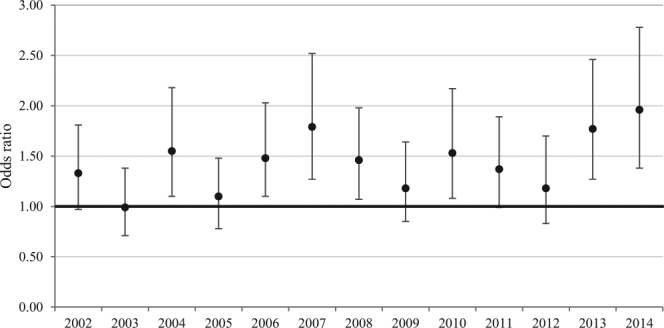
Risk-adjusted mortality odds ratios by year for females vs males.

Table 3 and Fig. 4 summarize sex differences in in-hospital mortality for each year of data. There was no discernable changing trend in odds of mortality among females versus males, with disparities persisting throughout the study period. Females had the highest odds of in-hospital mortality in 2014, the most recent year of data available (adjusted OR, 1.96; 95% CI, 1.38–2.78).

**Table 3:** Odds of rAAA mortality by year and gender.

Year	Male rAAA in-hospital deaths, No. (%)	Female rAAA in-hospital deaths, No. (%)	Odds Ratio, Females vs Males (95% CI)		
			Unadjusted	Adjusted^a^	P Value^b^
2002–2014	22,447 (53.5)	11,864 (41.9)	1.59 (1.49–1.71)	1.35 (1.24–1.47)	<0.001
2002	2,554 (48.6)	1,281 (61.0)	1.66 (1.33–2.06)	1.33 (0.97–1.81)	0.08
2003	2,495 (47.8)	1,007 (51.3)	1.15 (0.92–1.44)	0.99 (0.71–1.38)	0.95
2004	2,155 (46.1)	1,067 (55.5)	1.46 (1.15–1.84)	1.55 (1.10–2.18)	0.01
2005	2,114 (47.8)	1,033 (57.8)	1.50 (1.18–1.91)	1.10 (0.78–1.48)	0.66
2006	1,838 (43.5)	1,144 (60.0)	1.94 (1.54–2.45)	1.48 (1.10–2.03)	0.02
2007	1,667 (42.4)	850 (56.3)	1.75 (1.34–2.29)	1.79 (1.27–2.52)	0.001
2008	1,845 (41.2)	1,005 (50.7)	1.47 (1.15–1.88)	1.46 (1.07–1.98)	0.02
2009	1,600 (41.9)	813 (52.5)	1.53 (1.21–1.94)	1.18 (0.85–1.64)	0.33
2010	1,364 (35.9)	853 (52.4)	1.97 (1.53–2.52)	1.53 (1.08–2.17)	0.02
2011	1,370 (38.2)	764 (48.7)	1.46 (1.15–1.84)	1.37 (0.99–1.89)	0.06
2012	1,185 (35.3)	647 (42.6)	1.36 (1.02–1.82)	1.18 (0.83–1.70)	0.36
2013	1,210 (34.9)	760 (53.0)	2.10 (1.61–2.76)	1.77 (1.27–2.46)	0.001
2014	1,050 (32.2)	640 (49.0)	2.03 (1.54–2.68)	1.96 (1.38–2.78)	<0.001

^a^Adjusted for patient characteristics, comorbidities, and hospital characteristics.

^b^P values reported for adjusted ORs.

## Discussion

Previous research has shown disparities between sexes exist related to risk and outcomes of AAA. While females are generally protected from the development of AAAs, the ones that do develop behave more aggressively with higher growth and rupture rates, and are more likely to result in death^24,25^. However, to our knowledge, there has not been a multiyear comprehensive assessment of the disparities between females and males with regards to outcomes following hospitalization for rAAA. Using 13 years of data from the largest publicly available database of US inpatient stays, we found significant differences between females and males presenting to the hospital with a primary diagnosis of rAAA.

There were significant decreases in the rate of hospitalizations for rAAA among both females and males from 2002 through 2014. However, the hospital charges increased significantly for both sexes during the same time period. These rising charges were not due to increasing time spent in the hospital – the mean length of stay for males with rAAA was 11.3 days in 2002 and 9.5 days in 2014, compared with 9.4 days in 2002 and 6.6 days in 2014 for females (data not shown). Increasing hospital charges are most likely due to a variety of factors, including utilization of new technology and increasing drug prices.

We also investigated potential interaction effects between patient sex and time for dependent variables of adjusted mean charges and hospitalization rate – these analyses were conducted to investigate if the delta between sexes for each dependent variable changed significantly over time. The interaction between sex and time for mean hospital charges resulted in a P value of 0.100, though the interaction effect for hospitalization rate was <0.001. This suggests that while the rate of rAAA hospitalizations decreased over time for both sexes, the delta between the rate for males and that for females decreased significantly over time.

There were also significant decreases over time for both sexes in the proportion of patients dying in the hospital, though in each year females had a higher in-hospital mortality rate than males. After adjusting for potential confounders, we found that overall females had 35% higher odds of mortality than males despite having similar van Walraven Elixhauser comorbidity scores with males and similar perioperative complications. This confirms previous research showing that females with AAA tend to have disproportionately worse outcomes than men^14,25^. The United States Preventive Services Task Force (USPSTF) has historically not recommended screening for AAA in females regardless of risk factors, though, as of the writing of the manuscript, the USPSTF is considering a revised draft plan for AAA screening that would include asymptomatic adults in both sexes^26^. It is possible that if this plan is implemented, we may see a reduction in the mortality disparity between males and females with rAAA. Due to the fact that AAAs are often asymptomatic until they rupture, it is not unreasonable to assume that the prevalence is currently underdiagnosed. However, AAA screening can also potentially lead to the opposite problem of overdiagnosis. Most screen-detected AAAs are small (70% are <40 mm in diameter), which, while having low risk of rupture, can lead to other harms for some patients, such as psychological burden and unnecessary surgery^27^.

We performed separate analyses on sex disparities among those receiving EVAR and OAR. The proportion of rAAA patients who received EVAR increased substantially for both males and females, while the proportion receiving OAR decreased. We also examined the interaction between sex and time for both surgeries to investigate the delta between males and females. The interaction for EVAR yielded a P value of 0.062, while for OAR the interaction P value was 0.118. These results suggest that while clearly the gaps between sexes in terms of the proportions receiving EVAR or receiving OAR have widened and narrowed, respectively, the results were not quite statistically significant.

Females who received EVAR were significantly more likely to die than males, even after adjusting for potential confounders. Interestingly, this disparity in mortality occurred despite no significant differences in any of the perioperative complications studied. Females who received OAR were also significantly more likely to die than males, though the result just met our significance threshold of 0.05. However, among these patients, females were shown to have a significant protective effect in the odds of perioperative venous thromboembolism and bowel obstruction, and no significant differences were shown for the other perioperative complications.

Disparities in mortality between sexes persisted across the study period. We separately calculated adjusted odds of in-hospital death for females vs males in each year, and found that consistently females had higher risk than males. Despite decreasing mortality rates for both sexes, females have remained at higher risk of death compared with males.

There are many potential reasons for these outcome disparities, including differences in diagnosis and treatment rates or inherent anatomic dissimilarities. It is also possible that the lack of recommended one-time screening for AAA in females could be partially responsible. However, it is clear that greater focus is needed on improving rAAA outcomes among females, and additional research is necessary to determine potential underlying pathophysiological explanations for outcome disparities between sexes.

While this study provides valuable information on disparities in outcomes for rAAA between sexes, there are some important limitations that warrant further examination. The NIS database is an administrative data set originally generated for billing purposes and is vulnerable to coding errors and incomplete data. We analyzed 13 years of data, consisting of over 75,000 hospitalizations for rAAA, which should diminish the effect of any potential coding errors. Similarly, due to the nature of administrative data we were unable to discern information on the pathophysiology of each patient’s AAA, including diameter of the ruptured vessel, vessel tortuosity, and site of rupture (intraperitoneal cavity or retroperitoneal cavity). Each of these variables may influence perioperative complications or in-hospital mortality and may have an effect on whether a patient is an appropriate candidate for EVAR or OAR. Finally, the NIS is restricted to in-hospital data and does not provide information on long-term outcomes.

Despite these potential limitations, this study has some notable strengths. Using 13 years of data from the largest publicly-available all-payer inpatient database in the United States, we conducted the first comprehensive study to focus on outcome disparities in hospitalizations for rAAA between females and males. Future research could focus on conducting similar analyses in the emergency department setting, which in conjunction with the results of this study would provide a complete picture of the clinical and economic characteristics of rAAA patients in the ED and hospital.

## Conclusions

To our knowledge this is the first comprehensive investigation focusing specifically on sex disparities in hospitalizations for rAAA. We demonstrate that, while overall the incidence and mortality of rAAA have improved over time for both sexes, females remain at higher risk of in-hospital death than males. This risk disparity persisted throughout the study period, despite females and males having similar van Walraven Elixhauser comorbidity scores and similar perioperative complications. Females were also less likely to undergo rAAA repair than males, and among patients who did receive either EVAR or OAR procedures, females were more likely to die in the hospital.
